# Different Ecological Niches for Ticks of Public Health Significance in Canada

**DOI:** 10.1371/journal.pone.0131282

**Published:** 2015-07-01

**Authors:** Vanessa Gabriele-Rivet, Julie Arsenault, Jacqueline Badcock, Angela Cheng, Jim Edsall, Jim Goltz, Joe Kennedy, L. Robbin Lindsay, Yann Pelcat, Nicholas H. Ogden

**Affiliations:** 1 Groupe de Recherche en Épidémiologie des Zoonoses et Santé Publique, Faculté de Médecine Vétérinaire, Université de Montréal, Saint-Hyacinthe, Québec, Canada; 2 Département de Pathologie et Microbiologie, Faculté de Médecine Vétérinaire, Université de Montréal, Saint-Hyacinthe, Québec, Canada; 3 Office of the Chief Medical Officer of Health, New Brunswick Department of Health, Fredericton, New Brunswick, Canada; 4 Environment Canada, Ottawa, Ontario, Canada; 5 Jim Edsall Insect Identification Services, Dartmouth, Nova Scotia, Canada; 6 New Brunswick Department of Aquaculture, Agriculture & Food, Fredericton, New Brunswick, Canada; 7 New Brunswick Natural Resources, Fredericton, New Brunswick, Canada; 8 Public Health Agency of Canada, National Microbiology Laboratory, Winnipeg, Manitoba, Canada; 9 Public Health Agency of Canada, Laboratory for Foodborne Zoonoses, Saint-Hyacinthe, Québec, Canada; University of Kentucky College of Medicine, UNITED STATES

## Abstract

Tick-borne diseases are a growing public health concern as their incidence and range have increased in recent decades. Lyme disease is an emerging infectious disease in Canada due to northward expansion of the geographic range of *Ixodes scapularis*, the principal tick vector for the Lyme disease agent *Borrelia burgdorferi*, into central and eastern Canada. In this study the geographical distributions of Ixodid ticks, including *I*. *scapularis*, and environmental factors associated with their occurrence were investigated in New Brunswick, Canada, where few *I*. *scapularis* populations have been found to date. Density of host-seeking ticks was evaluated by drag sampling of woodland habitats in a total of 159 sites. *Ixodes scapularis* ticks (*n* = 5) were found on four sites, *Ixodes muris* (*n* = 1) on one site and *Haemaphysalis leporispalustris* (*n* = 243) on 41 sites. One of four adult *I*. *scapularis* ticks collected was PCR-positive for *B*. *burgdorferi*. No environmental variables were significantly associated with the presence of *I*. *scapularis* although comparisons with surveillance data in neighbouring provinces (Québec and Nova Scotia) suggested that temperature conditions may be too cold for *I*. *scapularis* (< 2800 annual degree days above 0°C [DD > 0°C]) across much of New Brunswick. In contrast, the presence of *H*. *leporispalustris*, which is a competent vector of tularaemia, was significantly (*P* < 0.05) associated with specific ranges of mean DD > 0°C, mean annual precipitation, percentage of clay in site soil, elevation and season in a multivariable logistic regression model. With the exception of some localized areas, temperature conditions and deer density may be too low for the establishment of *I*. *scapularis* and Lyme disease risk areas in New Brunswick, while environmental conditions were suitable for *H*. *leporispalustris* at many sites. These findings indicate differing ecological niches for two tick species of public health significance.

## Introduction

Ticks are vectors of bacterial, viral and protozoal pathogens of importance for human and animal health [1]. An increasing incidence of tick-borne diseases has been reported world-wide in recent years, which constitutes a critical concern for public and animal health [2, 3]. Emerging and re-emerging tick-borne diseases include Lyme disease, tularemia, Rocky Mountain spotted fever, ehrlichiosis, anaplasmosis, and babesiosis [4, 5]. Lyme disease, a bacterial disease caused by the spirochete *Borrelia burgdorferi*, is the most commonly reported tick-transmitted infection in temperate zones of the northern hemisphere, particularly in the United States and Europe [6–8]. The principal tick vector for *B*. *burgdorferi* in eastern and central North America is *Ixodes scapularis* [9]. Northward expansion of the geographic range of *I*. *scapularis*, from the United States into Eastern and Central Canada, is occurring and has resulted in an increased risk for Lyme disease in parts of southern Canada [10]. Associated with the range expansion of both *I*. *scapularis* and *B*. *burgdorferi*, the incidence of Lyme disease in Canada has risen markedly during recent years [10, 11]. The ticks are thought to be mostly introduced into Canada by migratory birds moving north in the springtime [12]. Mammalian hosts, such as the white-tailed deer *Odocoileus virginianus* Zimmermann, likely also play a role in dispersing ticks although mostly over short distances to nearby habitats [13]. Ticks carried by dispersing hosts, as well as the reservoir-competent hosts themselves, may be infected with *B*. *burgdorferi* and introduce the bacterium to locations where tick populations have become established resulting in the emergence of new Lyme disease risk areas [14].

Climate and landscape features as well as host densities affect the geographic distributions and densities of many tick species [15–19]. The ability of *I*. *scapularis* to survive and establish a self-sustaining, reproducing population in a new environment depends on these abiotic and biotic factors. Temperature seems to be an important limiting factor for the expansion of *I*. *scapularis* populations in Canada, via effects on rates of tick development from one life stage to another [20, 21]. Low temperatures increase the duration of developmental periods and consequently increase the duration of the life cycle and, as a result, the proportion of ticks that die before the cycle has been completed [20]. Rising temperatures associated with climate change are expected to permit or accelerate the northward spread of ticks such as *I*. *scapularis* [21–23]. In certain types of woodlands, the litter layer conditions provide a suitable environment for ticks to survive while undergoing development or seeking hosts, and protect them from unfavorable temperatures and humidity in winter and summer [24–26]. It is likely that a combination of interrelated factors including climate, soil types, drainage, aspect, elevation and the plant community affects how favourable the litter layer is for ticks [24, 27]. Finally, sufficient numbers of hosts need to be present for the ticks to survive as they are obligate parasites. For example white-tailed deer are key hosts for adult *I*. *scapularis* and densities of >7 per km^2^ have been considered essential for tick persistence [28, 29].

In 1998, *I*. *scapularis* in Canada was restricted to Long Point on the shores of Lake Erie, in Ontario [30]. More recently, established *I*. *scapularis* populations have been identified in multiple areas of southern Québec, Ontario, Manitoba, Nova Scotia and New Brunswick [11]. In New Brunswick, field surveillance campaigns in 2008 and 2010 revealed sites with *I*. *scapularis* ticks only in the Saint-John area. Subsequently, another localised area where *I*. *scapularis* ticks occur was found on Grand Manan Island. Passive tick surveillance data have suggested that *I*. *scapularis* populations may be becoming established in other areas in New Brunswick [14]. In eastern Canada there is little information on other tick vectors of Lyme disease (e.g. *I*. *dentatus*) [31] or tick species such as *Dermacentor variabilis* and *Haemaphysalis leporispalustris* that may be vectors of tick-borne diseases of public health importance such as tularaemia [32]. The present study was undertaken to i) evaluate the extent to which populations of *I*. *scapularis* and other tick species of public health importance may have become established in New Brunswick; and ii) to better understand the environmental determinants of their niches.

## Materials and Methods

### Site selection

A cross-sectional study was conducted between May and September 2014 to determine the presence of *I*. *scapularis* in New Brunswick and assess occurrence of other exophilic tick species. The study locations were selected to maximise our knowledge of the current distribution of *I*. *scapularis* and other ticks while encompassing enough study sites to provide information on the suitability of a variety of woodland types within four lowland ecoregions of New Brunswick (Fundy Coastal, Valley Lowlands, Eastern Lowlands and Grand Lake Lowlands as defined by New Brunswick Department of Natural Resources [33]). The number of sites per ecoregion (totalling > 140 sites for the whole study) was determined to detect differences in prevalence of tick-positive sites amongst ecoregions of 0 versus 0.25 with 80% power and alpha of 0.05. The algorithm for site selection was 1) selection of ecoregion as described above; 2) selection of woodlands (all types) of a minimum size of 200 x 200 m; 3) random selection of 280 sites from these woodlands; 4) removal of sites with no road access and within 50 m of any water body or wetland; 5) identification of cadastres to obtain owner information; and 6) final selection based on securing permission to visit the sites from the land owners where this was possible. Selected sites comprised woodlands on crown land which is open access, and private woodlands to which non-motorised access is not restricted in New Brunswick, although permissions were sought in most cases. No sites involved protected areas that would require permits for sampling and ethical or other permits were not required for collecting ticks by drag sampling.

### Tick collection

Each site was sampled once by one field operative. Sampling was conducted within a 10,000 m^2^ grid (200 m X 50 m). The collection of host-seeking ticks was carried out by dragging a 1-m^2^ flannel through vegetation over twenty 50 m transects with a pause every 10 m to inspect the flannel for ticks. GPS locations were collected at the start of field sampling. Ticks of any species collected were placed into tubes containing filter paper moistened with sterilised water and transported by courier to the National Microbiology Laboratory (NML) in Winnipeg where tick species were identified and any *I*. *scapularis* ticks tested by PCR for the tick-borne pathogens *Borrelia burgdorferi*, *B*. *miyamotoi*, *Anaplasma phagocytophilum* and *Babesia microti* [34].

### Environmental determinants of tick occurrence

Environmental variables for each study site were obtained from two sources, data collected at the site at the time of visit and data obtained from georeferenced databases.

#### Environmental data collected at each site visit

At each site during the visit, environmental data collected were i) Elevation aspect (categorized as hill, slope or level table), ii) Soil moisture characteristic (wet, moist, fresh or dry), iii) Soil texture composition (the percentage of soil comprising loam and clay on a scale of 0–100% using the methodology described in Lee et al. [35] and used in previous studies on environmental determinants of tick survival [36]), and iv) Dominant tree species within each site [35]. On the basis of tree species, the habitat of each site was categorized with reference to the New Brunswick forest classifications scheme [33] as 0 (woodlands of moist but well-drained fertile lowland soils—climax forest/closed canopy/shade tolerant), 1 (woodlands of well drained sandy soils), 2 (woodlands of wetlands/swamp) or 3 (woodlands comprising shade intolerant and/or pioneer species indicating disturbed woodlands).

#### Georeferenced environmental data

The following environmental features were extracted using ArcGIS version 10.2.2 (ESRI, Redlands, CA): i) Site elevation from Canadian Digital Elevation Model (CDEM) which has a geographic resolution of 20 m (Government of Canada, Natural Resources Canada, Earth Sciences Sector, Mapping Information Branch, GeoAccess Division, 2010, www.geogratis.gc.ca/site/eng/extraction); ii) Forestry data from the Digital Forest Resource Inventory data (2012) of the Natural Resources of New Brunswick. A circular buffer of 200 m was delineated around each site GPS point and the dominant tree species and the mean values of elevation were extracted for each buffer. The sites were classified into the same four classifications as described for the site-observed tree data using the dominant tree species in the buffer.

Land surface temperature (LST) for each site was derived from MOD11C3, a remotely-sensed Moderate Resolution Imaging Spectroradiometer (MODIS) satellite data, from 2009 to 2014, with a spatial resolution of 250 m. The monthly mean temperature above 0°C (averaged from the daytime and nighttime passes) was multiplied by the number of days in that month and then cumulatively added for each month of the year to obtain an estimate of the annual cumulative degree-days > 0°C (DD > 0°C) at each site. The mean annual DD > 0°C from 2009 to 2014 was subsequently calculated. According to the results obtained from Ogden et al. [21], approximately 2800 DD > 0°C was considered the minimum threshold temperature conditions for the establishment of self-sustaining populations of *I*. *scapularis* ticks. For each site, the number of years from 2009 to 2014, when the temperature was suitable for *I*. *scapularis* (i.e. DD > 0°C equal to or above 2800) was calculated. This six-year period was selected because the duration of the *I*. *scapularis* lifecycle is two to three years in length [21], and we considered that the temperature conditions over two to three potential *I*. *scapularis* lifecycles prior to and including the study period were the most appropriate for considering the temperature suitability of the sites. Additionally, the temperature-variable basic reproduction number *R*_0_ of *I*. *scapularis* (the capacity of this species to reproduce given certain environmental conditions) was also estimated for each site location for each year using the *I*. *scapularis* population model described by Wu et al [37] and calibrated by the MODIS temperature data. The annual *R*_0_ of *I*. *scapularis* at each site was averaged for the 2009 to 2014 time frame.

The total annual precipitation was obtained for each year from 2009 to 2013 (the most recently available data) for the GPS position of each site using an interpolated grid at a spatial resolution of 10 km developed by the National Land and Water Information Service of Canada [38] and mean annual precipitation calculated.

Estimates of densities of white-tailed deer (*Odocoileus virginianus*) were derived from in-house models developed by the New Brunswick Natural Resources, which is based upon roadkill and harvest data for each wildlife management zone in New Brunswick. The deer density estimate for the wildlife management zone in which a site occurred was ascribed to that site.

### Statistical analyses

#### Analysis of environmental determinants

The prevalence of sites where ticks were found was determined for each tick species, with 95% exact confidence limits. Logistic regression models were built in SAS version 9.3 (SAS Institute Inc., Cary, NC) to explore the relationships between environmental variables and occurrence of each tick species identified in the study. The models also accounted for ecoregions and season (‘spring’ being May and June, ‘summer’ being July and August and ‘autumn’ being September). The season of each visit was included as this could possibly have an effect on the sensitivity of detection due to different tick instars being active in different seasons. Two explanatory variables, which were the number of years with a DD > 0°C equal to 2800 or above, and the mean R_0_, are meaningful only for *I*. *scapularis* ticks and were therefore not considered for other ticks. A list of all explanatory variables is presented in Table 1. The models employed maximum likelihood logistic regression except when the numbers of positive sites were low, or when no positive sites were obtained in one category of the predictor, in which case the exact inference method for logistic regression was employed, which is recommended for analyzing unbalanced or sparse binary datasets [39]. The assumption of linearity between each continuous variable and the log odds of the outcome was verified using graphical method with categorization [40] as well as by visual inspection of Lowess smoothed graphs produced in STATA (StataCorp, College Station, TX). When the linearity assumption of continuous variables was not respected, the variables were categorized using the 33^rd^ and 66^th^ percentiles as cut-offs. All explanatory variables with *P* values ≤0.10 from univariate analyses were considered eligible for the multivariable logistic regression analysis allowing for liberal inclusion of variables. Pairwise Pearson correlations among explanatory variables selected from univariate analyses were calculated on the continuous scale. If a strong correlation (r| > 0.7|) was detected only one variable was included in the full model based on the strongest biological plausibility. The final model was obtained using a backward selection procedure with *P*>0.05 as the rejection criterion. The goodness-of-fit of the model was assessed by applying the Hosmer-Lemeshow statistic test and the area under the Receiver Operating Characteristic (ROC) curve (AUC) was used as a measure of the predictive ability of the model.

**Table 1:** Descriptive statistics of explanatory variables and results from univariate analyses for occurrence of *I*. *scapularis* and *H*. *leporispalustris* at the 159 field sites visited during the study.

		*I*. *scapularis*		*H*. *leporispalustris*	
Explanatory variable	n	% pos	*P*-value	% pos	*P*-value
**On-site ecological data**					
Clay (%)			0.82		<0.01
< 20	25	4.0		4	
20-49	77	2.6		35.1	
> 49	57	1.8		22.8	
Humus (%)			0.83		0.65
< 50	29	3.5		31.0	
50-79	63	1.6		22.2	
> 79	67	3.0		26.9	
Humidity of soil			0.69		0.70
Dry	58	1.7		27.6	
Fresh	50	4.0		28	
Wet	51	2.0		21.6	
Aspect			0.05		0.94
Hill	17	0.0		29.4	
Slope	32	9.4		25	
Low	110	0.9		25.5	
Forest classification on-site^a^			0.51		0.93
Drain	45	4.4		28.9	
Moist	34	2.9		26.5	
Shade	49	0.0		24.5	
Wet	31	3.2		22.6	
**Environmental georeferenced databases**					
Forest classification with GIS^a,b^			NA^c^		0.19
Drain	64	4.7		29.7	
Moist	7	0.0		0.0	
Shade	35	2.9		22.9	
Wet	51	0.0		25.5	
Mean elevation (m)^d^			0.55		0.05
< 66.4	53	3.8		35.9	
66.4-144.8	53	0.0		15.1	
> 144.8	53	3.8		26.4	
Mean DD > 0°C ^e^			0.17		<0.01
< 2653.5	52	1.9		17.3	
2653.5-2739.3	54	0.0		18.5	
> 2739.3	53	5.7		41.5	
Mean R_0_^f^			0.17		NA
< 0.66	52	1.9		NA	
0.66-0.77	54	0.0		NA	
> 0.77	53	5.7		NA	
Number of years with DD > 0°C ≥ 2800^g^			0.81		NA
0	46	2.2		NA	
1	34	0.0		NA	
2-6	79	3.8		NA	
Mean annual precipitation (mm)^h^			0.22		<0.01
< 1180.8	52	0.0		11.5	
1180.8-1243.5	54	1.9		24.1	
> 1243.5	53	5.7		41.5	
Deer density^i^			0.20		0.20
<1.06	48	0.0		16.7	
1.06-2.05	59	1.7		28.8	
>2.05	52	5.8		30.8	
**Other**					
Ecoregion			0.14		0.74
Central Uplands	3	33.3		33.3	
Eastern Lowlands	50	0.0		24.0	
Fundy Coastal	9	0.0		44.4	
Grand Lake Lowlands	11	0.0		18.2	
Northern Uplands	2	0.0		0.0	
Valley Lowlands	84	3.6		26.2	
Season^j^			1.00		<0.01
Spring	42	2.4		2.4	
Summer	78	2.6		32.1	
Autumn	39	2.6		38.5	

^a^ Dominant tree species categorized as 0: woodlands of moist but well-drained fertile lowland soils—climax forest/closed canopy/shade tolerant, 1: woodlands of well drained sandy soils, 2: woodlands of wetlands/swamp or 3: woodlands comprising shade intolerant and/or pioneer species indicating disturbed woodlands

^b^ No tree species identified for 2 sites

^c^ No solution is provided because of degenerate estimates

^d^ Mean elevation inside a buffer zone with a radius of 200 m defined around the geographic position of each site.

^e^ Mean annual degree days above 0°C from 2009 to 2014.

^f^ Mean annual *R*_0_ from 2009 to 2014.

^g^ Number of years where the annual degree days above 0°C was 2800 or higher from 2009 to 2014.

^h^ Mean annual precipitation data from 2009 to 2013.

^i^ White-tailed deer population estimates for each wildlife management zone

^j^ Season when site was visited with ‘spring’ being May and June, ‘summer’ being July and August and ‘autumn’ being September.

To test the validity of threshold temperature conditions of 2800 of DD > 0°C for invasion and persistence of populations of *I*. *scapularis*, field surveillance data collected in this study on *I*. *scapularis* were combined with field surveillance data obtained mostly by drag sampling in recent years in Québec and Nova Scotia (see [41] for details). For each location where field surveillance has taken place, the number of years in the last six years (2009–2014) in which DD > 0°C was > 2800 was obtained from the MODIS data described above. ROC analysis in STATA was then conducted to assess the specificity and sensitivity of the number of years when DD > 0°C was > 2800 to predict the presence or absence of *I*. *scapularis* at surveillance sites. The positive and negative predictive values (PPV and NPV) for the presence of *I*. *scapularis* for the number of years DD > 0°C was > 2800 were calculated using the formulae below.

PPV=TP/(TP+FP)

NPV=TN/(TN+FN)

Where TP, FP, TN and FN are true positives, false positives, true negatives and false negatives, respectively. See S1 File for surveillance data collected or used.

#### Spatial analysis

Geographic patterns of tick populations could be due to environmental suitability for the ticks alone, but may be confounded by patterns of spread from a source location. This may be particularly pertinent for *I*. *scapularis* which is expanding its geographic range in Canada [42]. The following analyses explored the possibility that geographic patterns were associated with spread rather than environmental suitability alone. First, the presence of spatial clusters of positive sites for each tick species discovered was explored using the Kulldorff spatial scan test in SatScan version 9.2 [43]. The Bernoulli model was used with a maximum cluster size of up to 50% of the population at risk. The significance (*P* < 0.05) of clusters was determined through 999 Monte Carlo replicates. Spatial clusters of tick-positive sites could, however, be due to spatial clusters of key environmental variables, so for any spatial cluster we explored whether environmental variables significantly associated with tick presence also explained the spatial cluster using logistic regression in which site presence in the cluster was the outcome, and the environmental variable was the explanatory variable. Second, we undertook ‘clustering’ analysis (i.e. whether the distribution of positive sites was more clustered or dispersed than a random distribution for a range of distances), using the Ripley spatial K-function [44], performed in ROCR package version 1.0–5 in R [45]. In this analysis, the plot of the difference (*D*) between the *K*-function calculated for positive sites and the *K*-function calculated for negative sites against distance was evaluated against a hypothesis of complete spatial randomness.

## Results

### Tick collection

**Fig 1:**
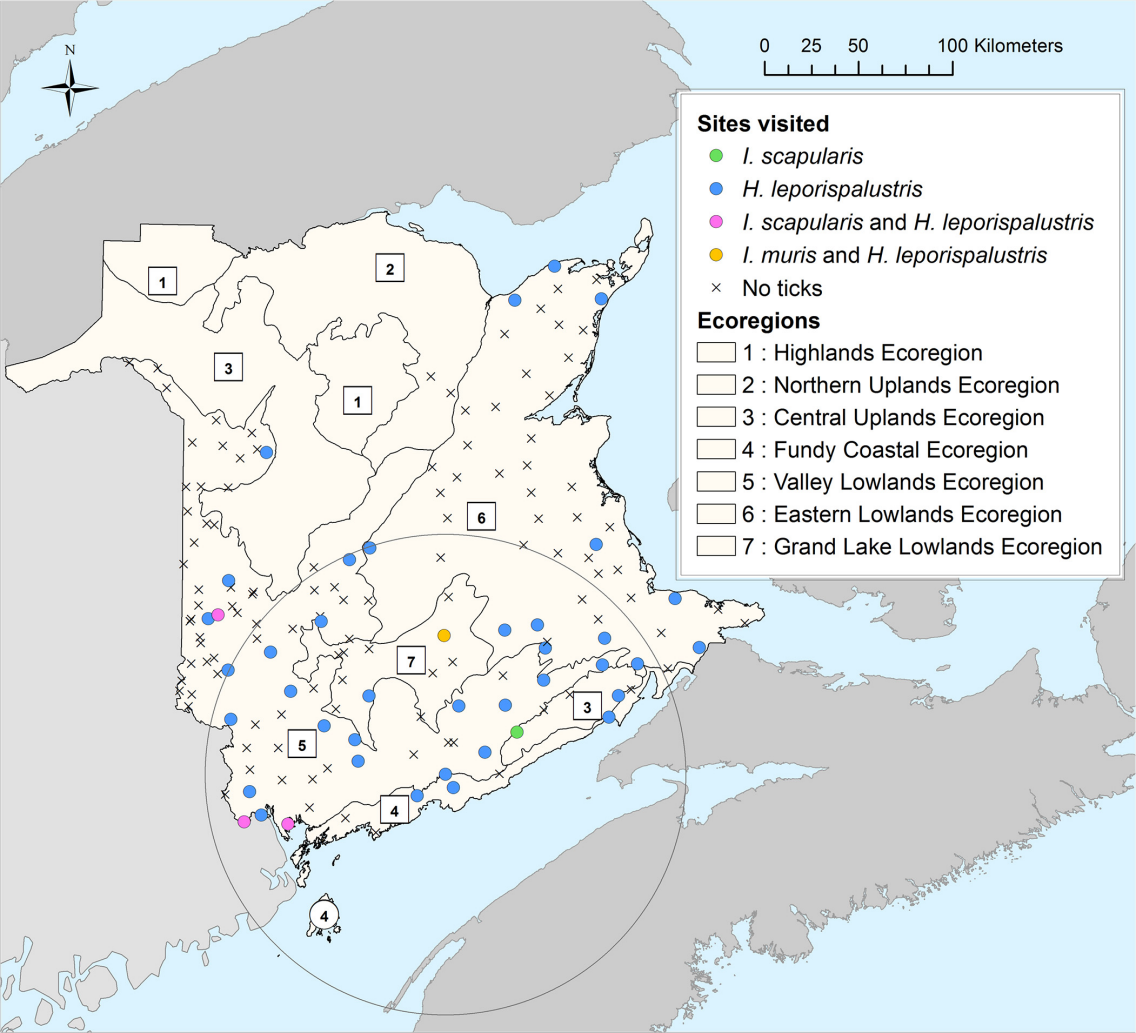
Distribution of sampling sites and tick-positive sites. Sites of occurrence of *I*. *scapularis* (green), *H*. *leporispalustris* (blue), *I*. *scapularis* and *H*. *leporispalustris* (pink) and *I*. *muris* and *H*. *leporispalustris* (yellow) are shown. The black circle indicates a significant cluster of sites where *H*. *leporispalustris* ticks were found. The two sites where *I*. *scapularis* were found in previous field studies are identified by red stars. The map was created in ArcGIS Version 10.2 (ESRI. Redlands, CA).

**Fig 2:**
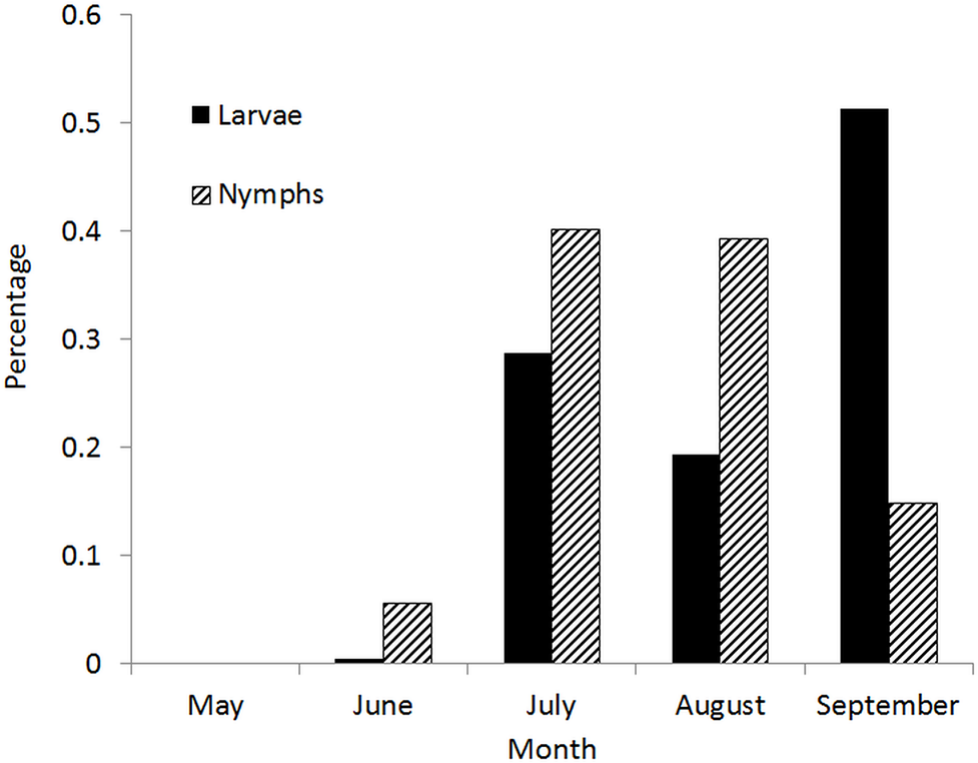
The seasonal activity of immature *H*. *leporispalustris*. The seasonality is expressed as the proportion of the total number of ticks of each instar collected in each month of the study.

A total of 159 sites that fulfilled the criteria for inclusion in the study were visited from May 27^th^ to September 24^th^ 2014. Sites were distributed over six ecoregions to obtain the sample size desired. This included the four initially targeted ecoregions as well as the Central Uplands and Northern Uplands ecoregions (Fig 1). One *I*. *scapularis* tick was found in each of three sites, of which two were adults and one was a larva. Additionally, two adult ticks were found on another site. This results in a prevalence of 2.5% of sites with *I*. *scapularis* ticks (exact 95% confidence interval [CI] = 0.7%-6.3%). A total of 243 *H*. *leporispalustris* were found on 41 sites with a range of 1 to 67 ticks per site, which corresponds to 25.8% of all sites visited (95% CI = 19.2%-33.3%). More than 90% of these *H*. *leporispalustris* ticks were larvae; the remainder were nymphs. In addition, one *Ixodes muris* nymph was detected on one site. Details on the numbers of ticks collected are presented in Table 2 and the locations of tick collection are shown in Fig 1. The adult *I*. *scapularis* were found in June, July and August, and the larval *I*. *scapularis* was found in September. Larval and nymphal *H*. *leporispalustris* were collected from June to September. For nymphal *H*. *leporispalustris*, a unimodal pattern of seasonal activity peaking in July-August was observed, and while peak activity of larvae occurred in September, a second peak occurred in July suggesting a bimodal seasonal pattern of activity (Fig 2).

**Table 2:** Numbers of ticks of each species and instar collected during the study. Note that all adult ticks were males.

		Number of ticks		
Tick species	Larva	Nymph	Adult	Number of positive sites
*I*. *scapularis*	1	0	4	4
*I*. *muris*	0	1	0	1
*H*. *leporispalustris*	223	20	0	41

One of the four *I*. *scapularis* adult ticks collected was positive for *B*. *burgdorferi*. PCR was not performed on the larval *I*. *scapularis* since the transmission of *B*. *burgdorferi* from female to their larval progeny does not seem to occur [46]. DNA of *B*. *miyamotoi*, *A*. *phagocytophilum* and *B*. *microti* were not detected in the four *I*. *scapularis* adult ticks.

### Environmental determinants of tick occurrence

Statistical models were developed for both *I*. *scapularis* and *H*. *leporispalustris*, with the exact inference method for logistic regression being employed for *I*. *scapularis* due to the sparse data. None of the continuous variables respected the linearity assumption and they were therefore categorized (Table 1). No variables were significantly associated with the presence of *I*. *scapularis* at the *P*<0.05 level of significance.

On univariable analyses for *H*. *leporispalustris*, five variables were significant at the *P* < 0.01 level, which were level of clay, mean elevation, mean DD > 0°C, mean annual precipitation and season (Table 1). Collinearity amongst these variables was not detected. All five predictors were retained in the final model after the backward selection procedure. Results from the full model are detailed in Table 3. The Hosmer-Lemeshow test of goodness of fit for the resulting model was not significant (*P* = 0.54) which indicates that the model fit the data well. Additionally, the AUC was 0.88 suggesting a high predictive ability of the model. Sites with higher annual DD>0°C (> 2739.3) were at greater risk of *H*. *leporispalustris* compared to sites with medium (2653.5–2739.3) and low (< 2653.5) annual DD>0°C. The range of mean annual DD>0°C at *H*. *leporispalustris*-positive sites was 2461 to 3003, with mean and median values of 2733 and 2746, respectively.

**Table 3:** Final multivariable logistic regression model for predicting occurrence of *H*. *leporispalustris*.

		Odds ratio		
Explanatory variables	Estimate	(95% CI)	*P*-value	Global *P*-value
Clay				<0.01
20–49 vs > 49	15.36	(3.32, 71.19)	<0.01	
20–49 vs < 20	4.30	(0.21, 86.55)	0.34	
< 20 vs > 49	3.58	(0.16, 81.69)	0.42	
Mean elevation				<0.01
< 66.4 vs 66.4–144.8	11.83	(2.45, 57.02)	<0.01	
> 144.8 vs 66.4–144.8	6.17	(1.43,26.62)	0.01	
< 66.4 vs > 144.8	1.92	(0.56,6.52)	0.30	
Mean annual DD > 0°C				0.02
> 2739.3 vs < 2653.5	6.48	(1.73, 24.34)	0.01	
> 2739.3 vs 2653.5–2739.3	3.67	(1.05, 12.90)	0.04	
2653.5–2739.3 vs < 2653.5	1.76	(0.47, 6.64)	0.40	
Mean annual precipitation (mm)				0.02
> 1243.5 vs < 1180.8	6.61	(1.68, 25.97)	0.01	
> 1243.5 vs 1180.8–1243.5	2.49	(0.83, 7.46)	0.10	
1180.8–1243.5 vs < 1180.8	2.65	(0.69, 10.13)	0.15	
Season				<0.01
Autumn vs Spring	160.02	(8.78, Infinity)	<0.01	
Summer vs Spring	24.43	(1.92, 311.39)	0.01	
Autumn vs Summer	6.55	(1.59, 26.91)	0.01	

Hosmer and Lemeshow goodness-of-fit test χ^2^ = 6.01, d.f. = 7, *P* = 0.54, AUC = 0.88.

**Fig 3:**
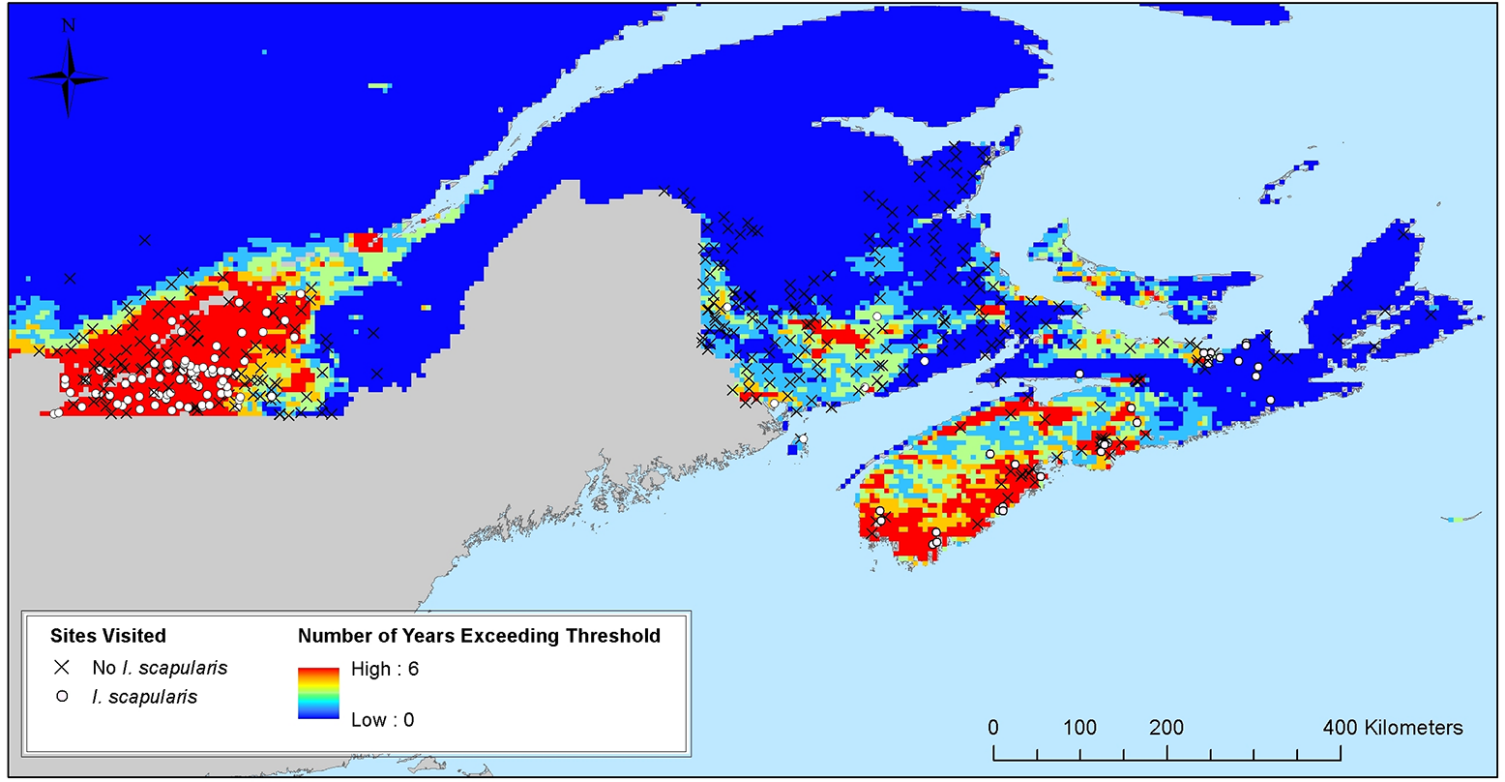
Relation of temperature conditions and results of field surveillance for *I*. *scapularis* in eastern Canada. The map shows the number of years from 2009–2014 in which DD > 0°C was greater than the model-based threshold for *I*. *scapularis* population survival obtained for southeastern Quebec and the Maritime provinces (New Brunswick, Prince Edward Island and Nova Scotia) from MODIS satellite data at a spatial resolution of 250 m. Sites where field surveillance for *I*. *scapularis* has occurred are indicated by circles (for locations where *I*. *scapularis* were found) and crosses (for locations where *I*. *scapularis* were not found). The map was created in ArcMap 10.2.2 (ESRI. Redlands, CA).

Data on *I*. *scapularis* surveillance conducted from 2007 to 2012 were available from 87 sites in Nova Scotia (where *I*. *scapularis* was found at 32) and 213 sites in Québec (where *I*. *scapularis* was found at 101), as well as two known localised areas where *I*. *scapularis* populations occur in New Brunswick, in addition to the 159 sites investigated in this study. Therefore there were data from a total of 461 sites for ROC analysis (Fig 3). As shown in Fig 3, sites in New Brunswick were colder than sites in Nova Scotia or Québec with only 20% (33/162) of sites in New Brunswick having experienced > 2800 DD > 0°C in more than one year from 2009–2014, compared to 70% (61/87) of sites in Nova Scotia and 90% (191/213) of sites in Québec. *Ixodes scapularis* were found at 37% (32/87) of sites in Nova Scotia and 47% (101/213) of sites in Québec. The overall area under the ROC curve was 0.78 (95% CI = 0.74–0.82) and details of the ROC analysis results are shown in Table 4. The sensitivity of detection of *I*. *scapularis*-positive sites was high using the criterion of multiple years in which DD > 0°C was > 2800, reaching values > 90%. *Ixodes scapularis* were found at only 3 of 77 sites at which temperature conditions did not reach > 2800 DD > 0°C in at least one year. Specificity was, however, comparatively low (as low as 17%), although this would be consistent with southeastern Canada being a zone of *I*. *scapularis* emergence where not all climatically suitable habitats would be expected to be occupied yet by tick populations, resulting in ‘false negative’ locations in the ‘gold standard’ field surveillance data. Consistent with this, the criterion of one or more years with DD > 0°C > 2800 had high (near or over 90%) negative predictive value for *I*. *scapularis*, while positive predictive value was considerably lower, consistent with climatically suitable sites not yet being occupied by *I*. *scapularis* populations (Table 4).

**Table 4:** Results of ROC analysis of the sensitivity and specificity of the number of years from 2009–2014 in which DD > 0°C was > 2800 for predicting the presence of *I*. *scapularis*-positive sites in Québec, Nova Scotia and New Brunswick using surveillance data collected in this and in previous studies.

Number of years DD > 0°C was > 2800	Sensitivity	Specificity	% sites correctly classified	PPV	NPV
1 or more	97.84%	17.65%	41.77	33.8	95.0
2 or more	95.68%	31.27%	50.65	37.4	94.4
3 or more	90.65%	50.77%	62.77	44.2	92.6
4 or more	85.61%	61.3%	68.61	48.8	90.8
5 or more	79.86%	68.42%	71.86	52.1	88.8
All 6	74.1%	74.92%	74.68	55.9	87.0

PPV = positive predictive value and NPV = negative predictive value expressed as a percentage.

### Spatial analysis

No spatial clusters of positive sites for *I*. *scapularis* were detected (relative risk [RR] = 38.75, *P* = 0.24, Fig 1). A cluster was however detected for *H*. *leporispalustris* (RR = 3.47, *P* = 0.012) with a radius of 127.96 km, containing 75 sites in the south of New Brunswick (Fig 1). An exact regression logistic model was developed to predict whether *H*. *leporispalustris*-positive sites were inside or outside the spatial cluster on the basis of environmental variables. Mean annual precipitation and mean DD > 0°C were found significant in the univariate analysis and both were subsequently retained in the final model (Tables 5 and 6). This suggested that the observed spatial cluster of positive sites in southern New Brunswick was likely associated with the spatial pattern of these two key environmental variables. In evaluating spatial autocorrelation of tick-positive sites using ‘clustering’ analysis, the *D* function remained within the envelope formed by upper and lower bounds of values consistent with an absence of significant clustering of tick-positive sites for both tick species [47].

**Table 5:** Results of univariate analyses for predicting whether *H*. *leporispalustris*-positive sites were inside or outside the spatial cluster.

Explanatory variables	n	% pos	*P*-value
Clay (%)			0.31
< 20	25	44.00	
20–49	77	53.25	
> 49	57	40.35	
Mean elevation			0.37
< 66.4	53	52.83	
66.4–144.8	53	49.06	
> 144.8	53	39.62	
Mean DD > 0°C			<0.01
< 2653.5	52	32.69	
2653.5–2739.3	54	35.19	
> 2739.3	53	73.58	
Mean annual precipitation (mm)			<0.01
< 1180.8	52	0.00	
1180.8–1243.5	54	44.44	
> 1243.5	53	96.23	
Season				0.11
Spring	42	35.71	
Summer	78	55.13	
Autumn	39	43.59	

**Table 6:** Final multivariable exact logistic regression model for predicting whether*H*. *leporispalustris*-positive sites were inside or outside the spatial cluster.

	Odds ratio			
Characteristics	Estimate	(95% CI)	*P* value	Global *P* value
Mean DD > 0°C				<0.01
> 2739.3 vs < 2653.5	10.89	(1.76, 125.03)	<0.01	
> 2739.3 vs 2653.5–2739.3	3.58	(0.94, 14.93)	0.06	
2653.5–2739.3 vs < 2653.5	3.12	(0.52, 34.43)	0.30	
Mean annual precipitation (mm)				<0.01
> 1243.5 vs < 1180.8	>999.99	(193.93, Infinity)	<0.01	
> 1243.5 vs 1180.8–1243.5	48.36	(8.49, 596.81)	<0.01	
1180.8–1243.5 vs < 1180.8	38.10	(7.80, Infinity)	<0.01	

## Discussion

*Ixodes scapularis* ticks were found in only 4 of 159 sites in this study, which suggests that populations of this tick are currently rare in New Brunswick and that risk from Lyme disease is mostly very low. Even on the sites where *I*. *scapularis* were collected, tick abundance is very low. These findings could be the result of the introduction of adventitious ticks dispersed by migratory birds or the existence of a recently established population with ticks present at very low density [12, 14]. An immature tick was found at one site, and multiple ticks (two adult males) were found at another, possibly suggesting that reproducing populations are present at these two sites. However, at two sites only one adult male tick was collected and at one of these sites annual DD > 0°C was not >2800 in any year from 2009 to 2014, which was an uncommon finding in our whole 461 site database of field surveillance. While in general detecting at least one *I*. *scapularis* by drag sampling suggests the presence of a reproducing tick population, the presence of only one adult tick could mean that the tick was an adventitious tick carried into the site from another location in Canada or the US by a migratory bird [48]. One of the two *I*. *scapularis* adult ticks found on the same site was PCR-positive for *B*. *burgdorferi*. This tick may have been an adventitious tick that acquired infection as an immature tick in its location of origin [48], or have acquired infection on the site and provide evidence of local transmission of *B*. *burgdorferi* by an established tick population, which would have greater implications for public health. Further study is required to confirm whether or not these ticks came from a reproducing population in the early phase of establishment when tick abundance would be expected to be low [14], or if these ticks were more likely adventitious ticks. To date we have found that drag sampling is equally sensitive at detecting *I*. *scapularis* throughout the May to October period, likely because the large adults active in spring and autumn are easy to see but of relatively low abundance, while much smaller nymphs and larvae active in late spring to early autumn are more difficult to see but more numerous than adults [48]. It would, however, be prudent to undertake longitudinal studies at these sites and include examination of hosts (particularly captured rodent), because use of the latter increases sensitivity of detection of tick populations especially for tick populations at low densities [48]. A conclusion on the presence or absence of reproducing *I*. *scapularis* populations could be more certainly made if drag sampling and rodent capture were used together in a longitudinal study [48].

**Fig 4:**
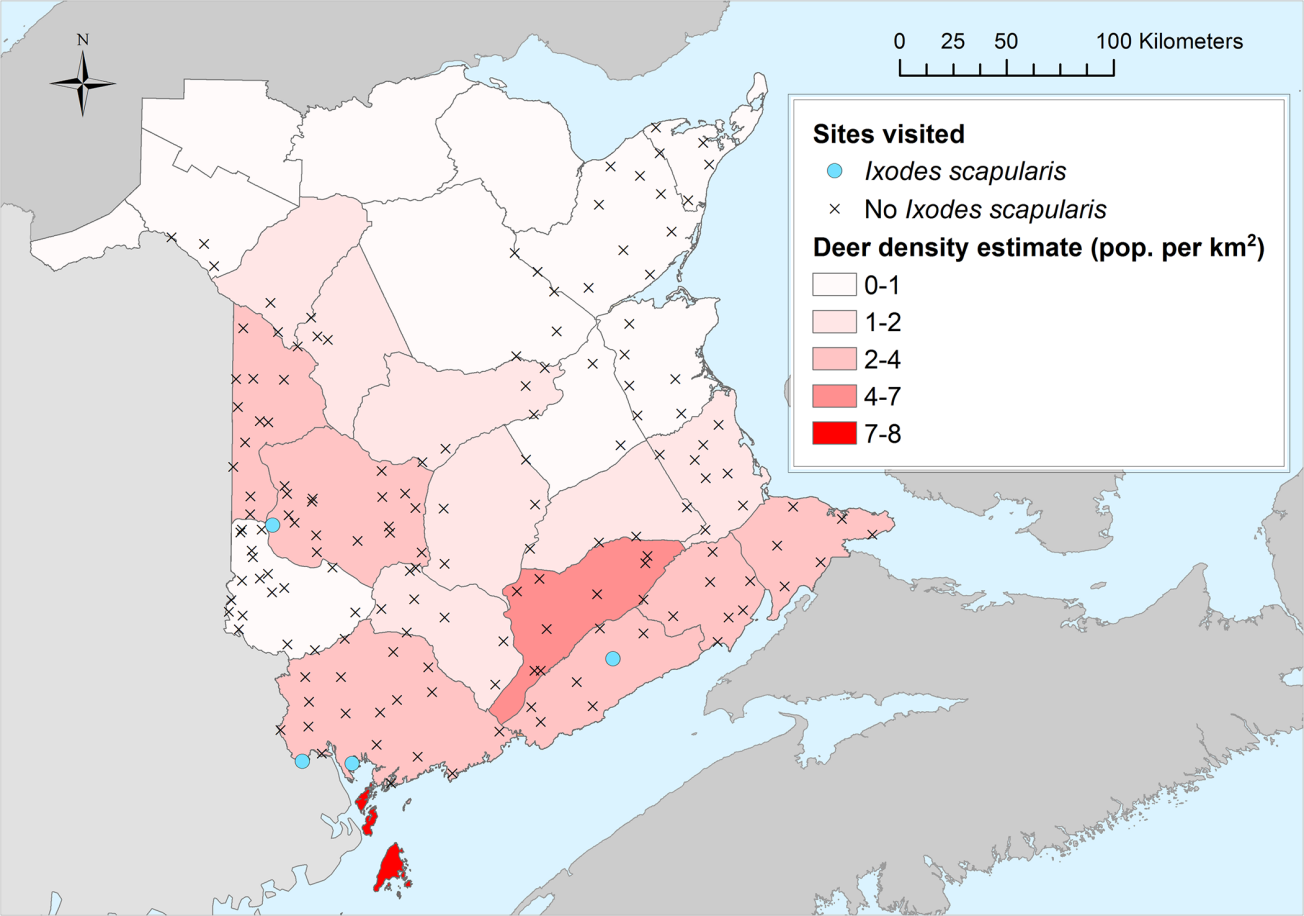
White-tailed deer densities. Estimated densities of white-tailed deer/km^2^ are shown for each wildlife management zone in New Brunswick for 2014. The map was created in ArcGIS Version 10.2 (ESRI. Redlands, CA).

Unsurprisingly given the small number of positive sites, no environmental factors were found to be significantly associated with the presence of *I*. *scapularis* and no spatial clusters of positive sites were detected. According to passive tick surveillance data, *I*. *scapularis* ticks are certainly dispersed into New Brunswick, and most of these likely come from the US by migratory birds or terrestrial hosts in the west of the province [48], and there is no reason to believe that the woodland types occurring in New Brunswick are intrinsically inhospitable to *I*. *scapularis*. Indeed, populations have established in some locations in New Brunswick (a location close to Saint-John and on Grand Manan Island), and *I*. *scapularis* has been shown to be able to establish in a wide range of coniferous, deciduous and mixed forest woodland types elsewhere in the United States and Canada [21, 25, 27, 49, 50]. Two factors may have been limiting however. First, the temperature conditions at the majority of sites were below, and the rest close to, the lower limits for *I*. *scapularis* population persistence obtained in previous studies [21] (Fig 3). There were stark differences in the proportions of surveillance sites positive for *I*. *scapularis* between Québec and Nova Scotia combined, and New Brunswick. These differences were associated with similarly stark differences between these regions in the numbers of recent years in which DD > 0°C were above the predicted threshold for *I*. *scapularis* population survival. Second, over most of the province, the estimated density of white-tailed deer was below the lower (7/km^2^) limit for *I*. *scapularis* population survival deduced in studies in neighbouring Maine [29] (Fig 4). Either or both of these factors may be limiting *I*. *scapularis* over much of New Brunswick. However, there are locations in southern New Brunswick, not investigated in this study, where temperature conditions have become favorable for *I*. *scapularis*, particularly the Saint John River system, South-West corner adjacent to Maine, the Moncton area and locations on the coast opposite to Prince Edward Island (Fig 3). These regions could possess microclimates where temperature conditions are suitable for *I*. *scapularis* establishment. Furthermore, there may be locations with microclimates too small to be detected by MODIS despite its high resolution. Unpublished road kill data also suggest that in localized areas, particularly in peri-urban areas, deer densities may exceed 7/km^2^ in the south of the province where temperature conditions are also more likely to be suitable for *I*. *scapularis*. Therefore it may be prudent to continue to undertake surveillance to detect emerging *I*. *scapularis* populations and Lyme disease risk in these regions.

In contrast to *I*. *scapularis*, *H*. *leporispalustris* ticks were found on many (> 25%) sites in New Brunswick and were abundant on most sites where they were found. *Haemaphysalis leporispalustris* is a vector of *Francisella tularensis*, the bacterial agent of tularaemia and this pathogen has been isolated from this tick species at sites elsewhere in Canada [51, 52] and from snowshoe hares in New Brunswick [53]. It is primarily a parasite of rabbits and hares although larvae and nymphs will also readily feed on birds [54] and it occasionally bites humans [55, 56]. The risk of it directly transmitting tularaemia to humans in New Brunswick is likely very low. However, it likely plays a role in *F*. *tularensis* transmission amongst wildlife reservoirs, thus indirectly contributing to risk to hunters who may acquire *F*. *tularensis* infection directly from infected wild animals [57]. The finding of *H*. *leporispalustris* on many sites served to validate the capacity of our field technique to detect questing exophilic ticks (and that negative results for *I*. *scapularis* were not false negative results) but also suggested that *I*. *scapularis* and *H*. *leporispalustris* may have very different ecological niches beyond simply differences in host species.

The results from the logistic regression modelling suggested that there was greater likelihood of finding *H*. *leporispalustris* on sites with high (> 2739) or medium (2653 to 2739) DD > 0°C compared to sites with low (< 2653.5) DD > 0°C. This is consistent with findings in California, where the presence of *H*. *leporispalustris* was correlated with the mean annual DD > 10°C [17]. However in contrast to *I*. *scapularis*, for which the ROC analysis of surveillance data supported an approximate minimum DD > 0°C of 2800 for persistence of populations, *H*. *leporispalustris* were found on sites with DD > 0°C as low as 2461 and the mean value of DD > 0°C of *H*. *leporispalustris*-positive sites was < 2800. Therefore, *H*. *leporispalustris* populations appear capable of surviving in a colder climate than *I*. *scapularis*. Higher precipitation (> 1243 mm) was associated with a greater likelihood of collecting *H*. *leporispalustris* compared to low precipitations (< 1181 mm) suggesting that a more humid environment may increase survival rates, and/or activity and detectability by drag sampling, of *H*. *leporispalustris*. In general more humid environments favour Ixodid tick survival and activity [58, 59]. Soil containing a medium proportion of clay (20% to 49%) was more associated with *H*. *leporispalustris* than soils with a high proportion of clay (>49%). The amount of clay in the soil may have an indirect relationship with the presence of *H*. *leporispalustris* by affecting other environmental qualities such as the litter layer and understorey plant communities that impact tick survival. High moisture levels in clay soils may promote growth of micro-organisms directly deleterious to tick survival [60]. *Ixodes scapularis* may, however, be more resistant than *H*. *leporispalustris* to clay soils [24]. The presence of *H*. *leporispalustris* was more likely on sites at low (< 66 m) and high (> 145 m) elevations compared to sites at intermediate elevations (66–145 m). Why this is the case is unclear when the model accounts for climate and associations with woodland and soil types and levels of moisture. However we speculate that this reflects effects of climatic conditions, vegetation composition or host density at a fine geographic scale. We accounted for the seasonality of the activity of *H*. *leporispalustris* and the observed pattern of activity (Fig 2) was consistent with other studies in which peak infestations of hosts by immature *H*. *leporispalustris* occurs in late summer [61, 62]. It has been suggested that adult *H*. *leporispalustris* feed on hosts in spring and the eggs they lay give rise to a peak of larvae in late summer while the spring peak may represent larvae that overwinter [61].

Our analysis of the spatial pattern of *H*. *leporispalustris*-positive sites revealed a significant spatial cluster in the south of the province but the occurrence of this cluster could be explained by the spatial pattern of temperature and rainfall conditions suitable for the tick. This idea was supported by the lack of spatial autocorrelation of *H*. *leporispalustris*-positive sites, i.e. sites were not significantly more likely to be *H*. *leporispalustris*-positive if they neighboured an *H*. *leporispalustris*-positive site. We interpret these findings as supportive of the idea that the spatial pattern of occurrence of *H*. *leporispalustris* in New Brunswick is due to the spatial pattern of an environment suitable for this tick, rather than providing evidence of recent spatial spread. Again this supports the idea that the climatological niche for *H*. *leporispalustris* is different to that of *I*. *scapularis* and perhaps has been less affected (at least in the geographic area under study here) to recent changes in climate. For *I*. *scapularis*, there has been evidence in previous studies for a spatial pattern of occurrence of *I*. *scapularis* associated with spread from one location to an adjacent location as well as effects of a warming climate [42, 63].

A single nymphal *I*. *muris* tick was collected; however *I*. *muris* ticks are more nidicoulous than *I*. *scapularis* and *H*. *leporispalustris* and rarely quest on vegetation [64]. That this tick was uncommonly found by drag sampling does not infer, therefore, that the tick is uncommon in New Brunswick. From 2007 to 2014, 66 submissions of *I*. *muris* were received by the authors as part of the passive tick surveillance in New Brunswick and 13 of these ticks were removed from humans. This verifies that *I*. *muris* will occasionally feed on humans and it is a competent, albeit inefficient, vector of *B*. *burgdorferi* [65] so cryptic transmission cycles associated with this tick, as can occur with other tick species (e.g. [31]), cannot be ruled out.

In conclusion, this study revealed that populations of *I*. *scapularis* ticks are currently uncommon in most of New Brunswick and this is most likely due to a combination of unsuitable or suboptimal conditions of temperature and abundance of key hosts (white tailed deer). Nevertheless, *I*. *scapularis* were found and our study suggests the occurrence of focal areas of possibly suitable environment and ongoing field surveillance targeted to sentinel at-risk locations, such as peri-urban and suburban areas of southern New Brunswick, may be prudent. By evaluating the wider occurrence of *I*. *scapularis* in neighbouring Nova Scotia and Québec, the study provided empirical support for model-derived estimates of approximately 2800 DD > 0°C as being minimum temperature conditions required for this tick, thus further supporting projected distributions of the tick with climate change [42]. The study also revealed that the tick *H*. *leporispalustris* was widespread in southern New Brunswick and in contrast to *I*. *scapularis*, its populations are capable of surviving temperature conditions less than 2500 DD > 0°C and show little evidence of recent spread in New Brunswick. Therefore these two Ixodid tick species, which are vectors of several important zoonotic diseases, have different ecological niches underlining the need to assess the occurrence of vectors and risk from vector-borne zoonoses separately according to their individual ecologies.

## Supporting Information

S1 File Surveillance data collected or used in the study. These data include locations of study sites and results of surveillance (presence/absence of tick species) as well as explanatory variables used in the final multivariable logistic regression model for predicting occurrence of *H*. *leporispalustris*.
(XLSX)
